# Altered brain activation and functional connectivity in working memory related networks in patients with type 2 diabetes: An ICA-based analysis

**DOI:** 10.1038/srep23767

**Published:** 2016-03-29

**Authors:** Yang Zhang, Shan Lu, Chunlei Liu, Huimei Zhang, Xuanhe Zhou, Changlin Ni, Wen Qin, Quan Zhang

**Affiliations:** 1 Department of Radiology and Tianjin Key Laboratory of Functional Imaging, Tianjin Medical University General Hospital, Tianjin 300052, China; 2 Department of Radiology, Tianjin Medical University Metabolic Diseases Hospital, Tianjin, 300060, China; 3 Department of Cardiology, Tianjin Medical University Metabolic Diseases Hospital, Tianjin, 300060, China

## Abstract

Type 2 diabetes mellitus (T2DM) can cause multidimensional cognitive deficits, among which working memory (WM) is usually involved at an early stage. However, the neural substrates underlying impaired WM in T2DM patients are still unclear. To clarify this issue, we utilized functional magnetic resonance imaging (fMRI) and independent component analysis to evaluate T2DM patients for alterations in brain activation and functional connectivity (FC) in WM networks and to determine their associations with cognitive and clinical variables. Twenty complication-free T2DM patients and 19 matched healthy controls (HCs) were enrolled, and fMRI data were acquired during a block-designed 1-back WM task. The WM metrics of the T2DM patients showed no differences compared with those of the HCs, except for a slightly lower accuracy rate in the T2DM patients. Compared with the HCs, the T2DM patients demonstrated increased activation within their WM fronto-parietal networks, and activation strength was significantly correlated with WM performance. The T2DM patients also showed decreased FC within and between their WM networks. Our results indicate that the functional integration of WM sub-networks was disrupted in the complication-free T2DM patients and that strengthened regional activity in fronto-parietal networks may compensate for the WM impairment caused by T2DM.

Type 2 diabetes mellitus (T2DM), a chronic metabolic disorder characterized by hyperglycemia due to insulin resistance, is growing in prevalence worldwide^1^. T2DM not only impairs metabolic-related bodily functions, but it is also associated with multidimensional cognitive deficits, such as deficits in execution, attention and memory^2^,^3^. Although cognitive decline may not be severe enough to affect the normal life quality of T2DM patients at an early stage^2^,^4^, compelling evidence suggests that T2DM can accelerate the aging process and increase the risk for dementia^5^,^6^. Recently, early detection and prevention have been considered the most promising strategies in coping with progressive dementia^7^; therefore, it is of great significance to identify the signs of early cognitive decline in T2DM patients before they develop dementia.

Neuroimaging methods can provide important insights into the neural mechanisms of cognitive impairment in patients with T2DM. For example, T2DM patients have been shown to exhibit distributed gray matter atrophy in the hippocampus, amygdala, and prefrontal and parietal cortices, and this atrophy might contribute to cognitive impairment^8^,^9^,^10^,^11^. T2DM is also frequently accompanied by brain vascular lesions, which have been associated with cognitive deficits^4^,^12^. Moreover, T2DM patients have been shown to exhibit alterations in regional spontaneous brain activity that are correlated with poor cognitive performance^13^. Finally, reduced resting-state functional connectivity (FC) has also been identified within the default mode network (DMN) of patients with T2DM, even in cases where brain structure and cognitive functions were not significantly impaired^14^. These studies indicate that neuroimaging techniques have the potential to provide early detection of brain impairment and cognitive deficits in T2DM patients.

Within the impaired cognitive domains, memory and executive functions are often involved at an early stage of T2DM^2^. Working memory (WM) is an important fundamental cognitive process for the temporary storage and manipulation of information in the brain, and it is crucially important for most higher-order cognitive functions^15^,^16^. WM impairments have been detected in T2DM patients^17^,^18^; however, the neural substrates underlying this impaired WM are still unclear. Several previous studies have investigated this issue using structural or functional magnetic resonance imaging (fMRI) techniques. For example, T2DM patients have been shown to exhibit gray matter loss in brain regions, which is associated with WM processing^8^,^9^, and two recent studies found brain activation changes in T2DM patients as they performed an n-back WM task^19^,^20^. However, these studies either failed to establish a direct link between neuroimaging phenotypes and WM^8^,^9^ or demonstrated contradictory results^19^,^21^. Furthermore, no previous studies have focused on the functional coupling of multiple WM brain regions, i.e., FC. Neural activity for WM is represented not only by the synchronization of clustered regional neurons (functional segregation) but also by the collaboration of multiple remote brain hubs (functional integration)^22^,^23^. Thus, the simultaneous detection of regional brain activation and functional coupling in WM networks under the performance of a specific WM task may help to elucidate the neurophysiological mechanisms underlying the cognitive deficits associated with T2DM.

Independent component analysis (ICA) is a promising statistical technique to reach the above-described goal. ICA is a powerful data-driven statistical technique that can be used to fully describe hidden task-related activation without a priori experimental model or the need for assumptions about the shapes of hemodynamic response curves^24^,^25^. Furthermore, ICA can be used to extract multiple independent functional components (also termed “networks”). The spatial patterns of blood oxygen level-dependent (BOLD) signal fluctuations are relatively unique among independent components, while the timecourses of BOLD signals are temporally coherent (or termed as FC) among voxels within each independent component. Thus, ICA is suitable for the detection of both regional activation patterns and FC patterns associated with task-related networks. Based on these merits, ICA has been successfully applied to WM-related studies of healthy humans^26^,^27^ as well as individuals presenting with psychiatric disease^28^,^29^.

In the current study, an ICA method was used to derive WM-related networks in fMRI data collected from a cohort of 20 complication-free T2DM patients and 19 matched healthy controls (HCs) during a block-designed digital 1-back WM task. Then, intergroup differences in both the activation and FC of the WM networks were compared. Associations between neuroimaging scalars and cognitive and clinical variables were also analyzed. Based on He’s study^20^ showing compensatory hyper-activation of WM related brain circuits in newly diagnosed complication-free T2DM patients, we hypothesized that the complication-free T2DM patients in our study may show similar hyper-activation in ICA-derived WM networks. Furthermore, because functional disconnectivity has previously been identified in several cognitive networks under resting-state in T2DM patients^14^,^30^, we predicted that FC within and between WM networks may also be impaired in our cohort of complication-free T2DM patients. Finally, we expected to discover significant associations between these neuroimaging phenotypes and WM performance and clinical variables in these patients.

## Results

### Demographic and behavioral results

**Figure 1:**
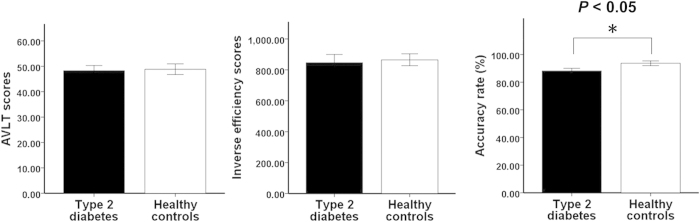
Intergroup comparisons of WM performance. T2DM patients show no statistical difference in either short memory scores of AVLT or 1-back inverse efficiency scores compared with the healthy controls except for a slightly lower 1-back accuracy rate. AVLT = auditory verbal learning test; WM = working memory.

The main demographic, clinical, cognitive information and WM task performance data for our cohort of T2DM patients and HCs are presented in Table 1. There were no significant differences in age, gender, education, or clinical variables between the two groups (*P* > 0.05). Compared with the HCs, the T2DM patients had significantly higher glycosylated hemoglobin (HbA1c) and fasting blood glucose (FBG) levels (*P* < 0.001). There were no significant differences in Auditory Verbal Learning Test (AVLT) short memory scores (*t* = −0.203, *P* = 0.840), or in inverse efficiency scores (IES) under 1-back WM conditions (*t* = −0.284, *P* = 0.778) between the two groups. The accuracy rate under 1-back WM conditions was slightly lower in the T2DM patients compared with the HCs (*t* = −2.189, *P* = 0.035, ES = −0.703) (Fig. 1).

### WM network spatial patterns

**Figure 2:**
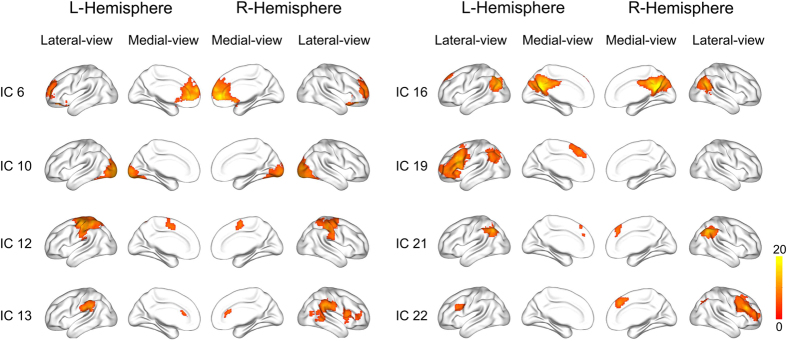
The eight WM-related networks derived from independent component analysis. Spatial structures were overlaid on surface brain maps. The components are numbered using the arbitrary order resulting from the IC estimation. The color bar represents the *t* value by one-sample *t*-test (*P* < 0.05, Family wise error corrected for multiple comparisons). IC = independent component; WM = working memory.

As shown in Fig. 2, among 23 automatically generated independent components (ICs), eight ICs were statistically related to the WM task. IC6 mainly contained the bilateral medial prefrontal cortex (MPFC). IC10 mainly included the middle and inferior occipital gyrus. IC12 mainly included the bilateral precentral, postcentral and paracentral gyrus. IC13 mainly included the bilateral supramarginal gyrus (SMG), the bilateral dorsal anterior cingulated cortex (dACC), the right posterior superior temporal gyrus (pSTG), the right inferior frontal gyrus (IFG) and the anterior insula (aINS). IC16 mainly included the bilateral precuneus/posterior cingulated cortex (PCC) and the angular gyrus (AG). IC19 mainly included the left middle frontal gyrus (MFG), the IFG, the aINS, the AG and the supplementary motor area (SMA). IC21 mainly included the bilateral intra-parietal sulcus (IPS) and the SMA. IC22 mainly included the left posterior IFG, the right MFG, the IFG and the SMA.

### Activation analyses of WM networks

**Figure 3:**
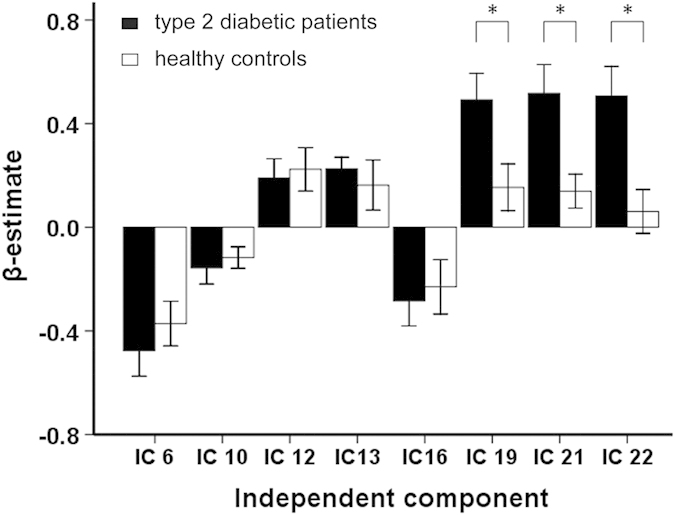
Intergroup comparison of brain activation amplitudes within each WM network. Asterisk represents significant difference in brain activation of a certain WM network between the T2DM patients and the healthy controls (*P* < 0.05, Bonferroni correction). IC = independent component; WM = working memory.

**Figure 4:**
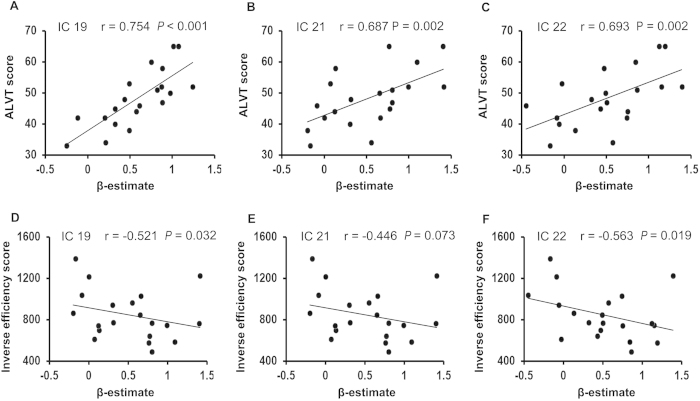
Correlation between activation strength (β-estimate) of each WM network and the behavioral performance in T2DM patients. (**A**–**C**) Correlation between β-estimate of each WM network (or IC) and short memory scores of AVLT; (**D**–**F**) Correlation between β-estimate and inverse efficiency scores of 1-back task. AVLT = auditory verbal learning test; IC = independent component; WM = working memory.

Compared with the HCs, the T2DM patients showed significantly higher activation in three fronto-parietal ICs after controlling for the effects of age, gender and education (IC19, *t* = 3.432, *P* = 0.001, effect size [ES] = 0.793; IC21, *t* = 2.901, *P* = 0.006, ES = 0.936; IC22, *t* = 3.115, *P* = 0.004, ES = 1.002; Bonferroni correction) (Fig. 3). To validate that these three ICs were activated by WM in both the T2DM patients and the HCs, we performed a traditional voxel-wise activation analysis. Based on this analysis, we found that the spatial distributions of WM-evoked activation in both the HCs and the T2DM patients was highly overlapped with that of the three ICs; moreover, the strength and spatial extent of WM-evoked activation in the T2DM group were stronger than those in the HCs groups (See Supplementary Figures S1 and S2). Furthermore, in the patients with T2DM, the activation amplitudes of IC19, IC21 and IC22 were significantly positively correlated with AVLT short-term memory scores; the activation amplitudes of IC19 and IC22 were significantly negatively correlated with the IES of the 1-back WM task, and a trend of negative correlation was shown between the activation amplitudes of IC21 and the IES of the 1-back WM task (Fig. 4). There was no significant correlation between the activation amplitudes and the accuracy rate in WM performance in the T2DM patients, and there was no significant correlation between the activation amplitudes and any WM indices in the HCs (*P* > 0.05). The correlations between activation amplitudes and FBG or HbA1c levels were not significant in the T2DM patients (*P* > 0.05).

### Functional connectivity analyses of WM networks

**Figure 5:**
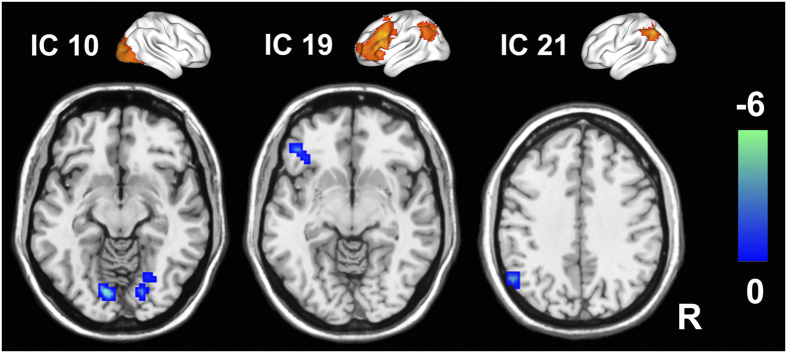
Brain regions showing decreased WM intra-network FC in T2DM patients (*P* < 0.05, AlphaSim correction). The top row shows the three ICs; the bottom row shows the regions with decreased FCs within corresponding ICs in T2DM patients. Color bar represents the *t* value. FC = functional connectivity; IC, independent component; WM = working memory.

**Figure 6:**
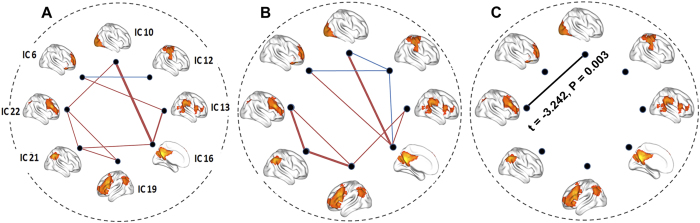
Group differences in FC between each pair of WM networks. (**A,B**) represents the inter-network FC in HCs and T2DM patients, respectively. (**C**) represents the intergroup difference in inter-network FC. Red lines represent positive FC, blue lines represent negative FC and black lines represent decreased FC of the T2DM patients compared with the HCs. FC = functional connectivity, HCs = healthy controls, WM = working memory.

Compared with the HCs, the T2DM patients exhibited significantly decreased FC in the bilateral lingual gyri of IC 10, the left ventral lateral prefrontal cortex (vlPFC) of IC19, the inferior parietal lobule (IPL) of IC 21 (*P* < 0.05, AlphaSim correction) (Table 2 and Fig. 5). Furthermore, compared with the HCs, the T2DM patients also showed significantly decreased FCs between IC22 and IC10 (*t* = −3.242, *P* = 0.003, ES = 0.646) (*P* < 0.05, uncorrected) (Fig. 6). There were no significant correlations between FCs in the WM intra-/inter- networks and clinical/cognitive variables in the T2DM patients.

## Discussion

In this study, we combined fMRI and ICA techniques to investigate changes in brain activation and FC in WM networks in complication-free T2DM patients as they performed a digital 1-back WM task. Significantly increased activation was identified in several WM-related fronto-parietal networks in the assessed T2DM patients, and the activation amplitudes of these WM networks were significantly associated with WM performance. In contrast, significantly decreased FC within the visual network (IC 10), the left fronto-parietal network (IC 19), and the posterior parietal network (IC 21) as well as decreased FC between the right fronto-parietal network (IC 22) and the visual network (IC 10) were shown in the complication-free T2DM patients. These inverse alterations in brain activation and FCs in WM networks may provide new insights into the neural mechanisms underlying WM impairment in T2DM patients.

### Increased fronto-parietal activation in working memory networks

Compared with the HCs, the T2DM patients demonstrated hyper-activation within three WM-related fronto-parietal components (IC19, IC21 and IC22). The fronto-parietal regions have been indisputably associated with WM in normal subjects^31^,^32^. Furthermore, impairments in fronto-parietal regions are usually evident in T2DM patients. For example, previous structure-imaging studies have demonstrated that gray matter and white matter in fronto-parietal regions are damaged in T2DM patients^8^,^33^, and reduced regional cerebral blood flow has also been found in the fronto-parietal lobes of T2DM patients^34^. Our results were consistent with a recent study showing increased activation in the bilateral frontal cortices of newly diagnosed, complication-free, middle-aged T2DM patients as they performed digital n-back WM tasks^21^. However, our findings were not consistent with another study demonstrating decreased activation in the frontal cortexes of T2DM patients as they performed a digital n-back task^19^. This discrepancy is probably due to the variable characteristics of the assessed T2DM patients. In Chen’s study^19^, for example, complications were not controlled for in T2DM patients, while in both He’s study^20^ and the current study, only T2DM patients with no typical complications were enrolled.

The observed fronto-parietal network hyper-activations during the performance of n-back WM tasks may indicate the presence of a compensatory mechanism for inefficient information processing in T2DM patients. According to the theoretical concept of the compensation-related utilization of neural circuits hypothesis^35^, an inverted U-shaped hypothesis is popularly accepted to describe when the brain copes with cognitive tasks. According to this hypothesis, an initially compensatory increase occurs in brain activation under increased cognitive loading followed by a decrease in brain activation, which never compensates for the increase in cognitive loading. Such inverted U-shape compensatory patterns have been reported in mild cognition impairment (MCI) and Alzheimer (AD)^36^ and healthy elders^37^ in WM studies. In our study, the poor WM performance in the T2DM patients may suggest that T2DM patients have difficulties in coping with the WM tasks and may require greater neuronal loading to accomplish the WM task compared with the HCs. Thus, the increased fronto-parietal network activation observed in these patients may be explained by a compensatory mechanisms that occurs in response to microscopic neuronal impairment at an early stage of T2DM^38^,^39^. Furthermore, significant correlations were found between the activation amplitudes of fronto-parietal networks in T2DM patients and WM performance, suggesting that patients with stronger fronto-parietal activation possess better WM abilities. As a result, these correlation results further supported our hypothesis of the presence of compensatory mechanisms of brain activation during WM processing in T2DM patients.

### Reduced functional connectivity in working memory networks

Our T2DM patients showed decreased intra-network FC in the bilateral lingual gyri of the visual network (IC 10), the vlPFC of the left fronto-parietal component (IC19) and the left IPL of the posterior parietal component (IC21). In addition, inter-network FC between the right fronto-parietal network (IC22) and visual network (IC10) were also decreased in T2DM patients.

Early studies reported that both the inferior frontal and the inferior parietal regions were involved in WM^40^. The vlPFC is closely related to both temporary information maintenance^41^,^42^,^43^ and comparison^44^, and is particularly critical for WM^31^,^45^. Recently, increased neural activity in the vlPFC was found in older adults compared with young adults during the performance of more complex tasks relative to a baseline; additionally, a higher BOLD signal in the vlPFC was shown to predict the efficiency of WM performance^46^. In diabetes, the vlPFC was considered to be vulnerably damaged^19^. IPL was frequently reported as playing a role in short-term memory storage which is an important content of 1-back working memory task^47^,^48^. In diabetes, decreased resting state FC in bilateral IPL was demonstrated^49^. Another two regions showing decreased intra-network FC in the T2DM patients were the bilateral lingual gyri which were revealed as having a neural correlation with WM^50^, and as being involved in briefly storing sensory information in WM task^51^,^52^. Thus, the above findings indicate that T2DM might impair the functional coupling of the fronto-parietal, bilateral parietal and visual networks during WM processing.

In addition, the current study showed that inter-network FC between the visual network (IC10) and the right fronto-parietal network (IC22) was also decreased in the T2DM patients. A previous positron emission tomography study showed that right fronto-parietal network was associated with sustained, and possibly selective, attention during rapid visual information processing in WM^53^. Visual cortex was suggested to receive top-down modulation from frontal and parietal areas^54^. Recently, Roelfsema *et al*.^55^ found that activity changes in visual areas during WM are likely due to top-down projections from higher associative cortices. In our study, the decreased FC between the right fronto-parietal network (specific for higher cognitive processing, such as execution control, attention and WM) and the visual network (specific for visual sensory processing) may suggest functional uncoupling between the two networks when dealing with stimulus-driven and top-down signal transformations during performing the WM tasks in T2DM patients.

In this study, we did not find any significant association between fMRI indices and FBG or HbA1c levels. This finding is not consistent with an early study that reported a significant positive correlation between HbA1c level and brain activation in WM regions in newly diagnosed T2DM patients^20^. It should be noted that although the patients enrolled by both He *et al*.^20^ and our group were free of complications, blood glucose control was quite different between the patient groups: in the present study, blood glucose was well controlled in enrolled T2DM patients, while in the study by He *et al*.^20^, the HbA1c and FBG levels of the enrolled T2DM patients were markedly higher than those of the healthy subjects and the T2DM patients assessed in the present study. Thus, the lack of correlation between fMRI scalars and blood glucose levels may indicate changes in the activation and functional coupling of WM networks that are directly associated with neuronal impairment in WM networks rather than with blood glucose level.

### Limitations

The main limitation of the current study was the inclusion of a relatively small sample size, which may degrade its statistical power. In addition, only a 1-back WM task was performed; thus, interactions between load effects and T2DM on WM network features need to be further clarified in future studies.

## Conclusion

To the best of our knowledge, this is the first study to simultaneously investigate changes in regional brain activation and FC in WM-related networks in complication-free T2DM patients using fMRI and ICA techniques. We found increased activation within the WM fronto-parietal networks of T2DM patients, and this activation was significantly correlated with WM performance. In contrast, decreased functional coupling was found both within and between WM networks in T2DM patients. Our results indicate that the functional integration of WM sub-networks was disrupted in the complication-free T2DM patients and that strengthened regional activity in fronto-parietal networks may compensate for the WM impairment caused by T2DM.

## Research Design and Methods

### Participants

Twenty T2DM patients from the endocrinology department of Tianjin Medical University General Hospital and 19 matched euglycemia HCs were recruited in this study. The following inclusion criteria were used for the T2DM patients: 1) diagnosed with T2DM according to the 2010 criteria of the ADA^56^; 2) no T2DM-related complications, including diabetic retinopathy, diabetic nephropathy, and diabetic foot; 3) no history of hypoglycemia during the last two years. The following exclusion criteria were used for both the T2DM patients and the HCs: 1) Mini-Mental State examination (MMSE) score < 27; 2) BMI ≥ 30 kg/m^2^; 3) psychiatric or neurologic disorders that could influence cognitive function; 4) cerebrovascular events, including transient ischemic attack and stroke; 5) history of alcohol or substance abuse; 6) family history of dementia; 7) hypertension and hyperlipidemia. Eighteen of the 20 enrolled T2DM patients controlled blood glucose using oral hypoglycemic agents (eleven with Metformin only, two with Acarbose only, four with both Metformin and Acarbose, one with both Acrbose and Glipizide) and five of the 18 patients were also under treatment with insulin.

All participants underwent physical examinations, including measurements of height, weight, and resting blood pressure. FBG, HbA1c, total cholesterol (TC), triglyceride (TG), high-density lipoprotein (HDL) and low-density lipoprotein (LDL) cholesterol levels were also recorded. In addition, a battery of neuropsychological tests was performed to assess each participant’s general mental status and other cognitive domains. Possible dementia was screened by the MMSE^57^. Anxiety and depressive symptoms were evaluated with the Self-Rating Anxiety Scale (SAS)^58^ and the Self-Rating Depressive Scale (SDS)^59^, respectively. Short-term memory was tested using the Chinese version of the AVLT^60^,^61^.

The protocol used for this study was approved by the Ethical Committee of Tianjin Medical University General Hospital, and all of the participants provided written informed consent according to institutional guidelines. The study methods were conducted in accordance with the approved guidelines

### Task design for fMRI

We did behavioral tests of 2-back WM task on first eight T2DM patients and found that six of them had difficulties in finishing a 2-back task (See Supplementary Table S1); thus a block-designed digit 0-back and 1-back WM task was performed during fMRI acquisition on following 19 T2DM patients whose data was used for analysis. Six 0-back and five 1-back blocks were alternately presented. Each 0-back block contained 11 trials (one instruction trial and ten digit trials); each 1-back block contained 12 trials (one instruction trial and 11 digit trials, the last 10 trails required responses). Each block started with a task instruction (2000 ms), after which a continuous stream of digits was presented in the center of a screen. The presentation time for each digit was 1000 ms, and followed with black screen for 1000 ms, and then the next digit was presented (Supplementary Figure S3). In the 0-back condition, the subjects were told to respond if the present digit was identical to the target ‘0’. In the 1-back condition, the subjects had to judge whether the present digit was identical to the previous digit. Each block had 50% targets trails that were presented randomly. The digits were displayed in white 40-point Arial font on a black background. E-Prime 2.0 software (Psychology Software Tools, Sharpsburg, Pennsylvania, USA) was used to generate stimuli and record response. Each stimulus was presented through video goggles that were equipped on the coil using VisualSystem (NordicNeuroLab, Bergen, Norway). Behavioral responses (accuracy ratio and average reaction time) were collected using a ResponseGrips system (NordicNeuroLab, Bergen, Norway). To eliminate possible speed accuracy trade-offs, IES was calculated by dividing the average reaction time by the accuracy ratio, which represents the corrected reaction time^62^,^63^.

### MRI data collection

MRI was performed using a 3.0-Tesla MR system (Discovery MR750, General Electric, Milwaukee, WI, USA). Before the fMRI experiment, conventional T2-weighted images (T2WI) were acquired to rule out the presence of visible brain lesions. T2WI were acquired using a fast spin echo sequence with the following parameters: repetition time (TR) = 3400 ms, echo time (TE) = 85 ms, flip angle (FA) = 90°, field of view (FOV) = 256 mm × 256 mm, matrix = 256 × 256, slice thickness = 3 mm, slice gap = 1 mm. BOLD fMRI data were acquired using a gradient-echo single-shot echo-planar imaging (GE-SS-EPI) sequence with the following parameters: TR = 2000 ms, TE = 45 ms, FOV = 220 mm × 220 mm, matrix = 64 × 64, FA = 90°, slice thickness = 4 m, slice gap = 0.5 mm; slice number = 32. The total acquisition volume was 126. Before each fMRI acquisition, ten dummy scans were performed to allow the fMRI signals to reach a steady state.

### Data preprocessing

All functional images were preprocessed using Statistical Parametric Mapping software (SPM8; http://www.fil.ion.ucl.ac.uk/spm/software/spm8). First, slice timing correction was performed to correct for the inter-slice time delay within each volume. Second, motion correction was estimated and corrected using rigid co-registration. Subjects who had head shift greater than 2.0 mm or rotation greater than 2.0° were excluded from the analyses. Third, images were spatially normalized into Montreal Neurological Institute (MNI) space using a standard EPI template provided by SPM8 and were resliced into a voxel-size of 3 mm × 3 mm × 3 mm. Finally, the data were spatially smoothed using a 6-mm full width at half-maximum Gaussian kernel.

### Independent component analyses

Group spatial ICA was applied using GIFT software (http://icatb.sourceforge.net/, version 2.0d) to decompose the preprocessed fMRI images into spatial ICs as follows. First, 23 ICs were automatically estimated using the minimum description length criteria^64^. After that, a two-step principal component analysis was used to decompose the fMRI timecourses of the whole brain voxels into 23 principal components. This analysis was followed by group-level IC estimation using an Informax algorithm^65^. The most stable estimation of ICs was achieved by re-running the ICA analysis 100 times using the ICASSO method. Then, a spatial-temporal algorithm was used to back reconstruct the subject-level ICs from the group-level ICs. The spatial-temporal regression algorithm was introduced as following equations:


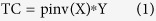



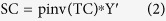


In which variable Y represents the group-level spatial components, and variable X represents the preprocessed fMRI timecourses of a certain subject, TC represents the temporal components of this subjects, and SC represents the spatial components of this subjects.

This step produced subject-level spatially IC maps (spatial components) as well as the featured time courses of these components (temporal components). Finally, the subject-specific spatial and temporal components were transformed into z-scores to create a normal distribution.

### Component selection

We first used spatial correlation analysis to exclude ICs that were not contributed by gray matter. To accomplish this, we employed the following steps: first, maximum probability maps (MPMs) of gray matter, white matter and cerebral blood flow were generated using the tissue prior templates provided by SPM. A certain tissue class (gray matter, white matter, or cerebral blood flow) was defined for each voxel in the MPM based on the maximum probability among the three tissue prior templates. Then, the values for the spatial IC map and the MPM were rearranged to include one vector for each map, and Pearson correlation analysis was performed to test the association between the vector of each spatial IC map and the vector of each MPM map. Finally, the highest spatial correlation coefficient of each IC was identified, and the corresponding tissue class was retrieved. Following these steps, 15 ICs were identified as gray matter components. We further excluded 2 ICs that belong to the cerebellum. Then, the remaining 13 candidate components were further tested by a first-level canonical general linear model (GLM) to clarify whether the featured time course of a specific component was statistically related to the WM task. Specifically, the time course for each component of each subject was regressed against the design matrix of the WM task using GLM and canonical hemodynamic response function (HRF). The resulting β-estimate of each component represents the activation of this component in response to WM loading (1-back versus 0-back) in the subject. The β-estimates were further assessed using a second-level random effect one-sample *t*-test to clarify whether the mean β-estimate of each component was statistically significant (*P* < 0.05, Bonferroni correction). In total, eight of the 13 candidate components were found to be statistically associated with the WM task and were chosen for further analysis (Fig. 2).

### WM-related intra-network and inter-network FC calculation

It should be noted that aforementioned subject-level spatial and temporal components were back-reconstructed using the full fMRI timecourses. Thus the components contained both 0-back and 1-back information. Because 0-back mainly reflects attention information but not the WM capacity, these subject-level components are not suitable for evaluate the WM-related FC changes in the T2DM. Thus, in order to obtain the spatial and temporal components that are specific for 1-back WM condition, we removed the timecourses that were abstained during the 0-back blocks from the raw fMRI data. We also corrected the HRF effect by delaying the task timing for 3 volumes (6 s). Then the remaining fMRI blocks were concatenate to form the timecourses that are specific for 1-back WM condition, and they were considered as the variable X in the dual-regression function. Finally the spatial components and temporal components were scaled using Z-score.

The value of each voxel within a spatial component reflects the temporal coherence between the BOLD timecourses of each voxel and its temporal component. Thus, we termed the value of the spatial component as the intra-network functional connectivity. We also calculated the inter-network functional connectivity as the Pearson correlation between the temporal components of each IC pair, which were further Fisher r-to-z transformed to satisfy parametric statistics.

### Statistical analyses

#### Activation analysis

Differences in global activation were tested by comparing the β-estimates of eight WM networks between the two groups using two-sample *t*-tests after controlling for the effects of age, gender and education (*P* < 0.05, Bonferroni correction). Finally, partial correlation coefficients were used to test the possible association between activation strength and clinical/cognitive variables after controlling for the effects of age, gender and education (*P* < 0.05).

#### Functional connectivity comparisons

A voxel-wise one-sample *t*-test was performed on the spatial components of the WM networks to recognize the spatial distribution pattern of the FC of each network. Multiple comparison corrections were made using family-wise error (FWE) correction (*P* < 0.05). The Cohen’s d effect size of each comparison was also calculated. Brain regions in each network showing statistically positive FC were binarized and used as mask for further intergroup comparisons. Intergroup differences in FC within eight networks were examined using two-sample *t*-tests after controlling for the effects of age, gender and education. Correction for multiple comparisons was performed using an AlphaSim algorithm, resulting in a corrected threshold of *P* < 0.05 at the cluster level (parameters: single voxel uncorrected *P* = 0.01, 1000 simulations, full width at half maximum = 6 mm, cluster connection radius r = 5 mm) and within the mask of the spatial distribution of each component. We further compared intergroup differences in FC between each pair of the eight WM networks using a two-sample *t*-test after controlling for the effects of age, gender and education (*P* < 0.05, uncorrected). The Cohen’s d effect size of each comparison was also calculated. Finally, partial correlation was used to assess possible associations between FC and clinical/cognitive variables after controlling for the effects of age, gender and education (*P* < 0.05).

#### Statistical analysis for demographic data

SPSS 21.0 (SPSS, Inc, Chiago.IL) was used to analyze demographic data. Shapiro-Wilk tests were performed to assess the distributions of demographic variables. Intergroup differences in demographic variables were tested either with Student’s *t*-test (normal distribution) or the Mann-Whitney *U*-test (non-normal distribution). Achi-squared (*χ*^2^) test was used to assess intergroup differences in gender. The significance level was set as *P* < 0.05.

## Additional Information

**How to cite this article**: Zhang, Y. *et al*. Altered brain activation and functional connectivity in working memory related networks in patients with type 2 diabetes: An ICA-based analysis. *Sci. Rep*. **6**, 23767; doi: 10.1038/srep23767 (2016).

## Supplementary Material

Supplementary Information

## Figures and Tables

**Table 1:** Demographics, clinical data, cognitive assessment.

	Diabetic patients (n = 20)	Healthy controls (n = 19)	statistics	*P*value
Age (years)	54.15 ± 8.78	51.58 ± 6.19	*t* = 1.052	0.300
Sex (M/F)	13/7	9/10	*x*^2^ = 1.232	0.341
Education (years)	12.15 ± 2.72	10.68 ± 2.26	*t* = 1.825	0.076
BMI (kg/m^2^)	24.99 ± 2.32	23.95 ± 2.75	*t* = 1.276	0.210
SBP (mmHg)	127.5 (110, 135)	125 (115, 135)	z = −0.682	0.496
DBP (mmHg)	80 (70, 85)	80 (70, 85)	z = −0.821	0.412
TC (mmol/L)	4.62 ± 0.52	4.58 ± 0.48	*t* = 0.225	0.823
TG (mmol/L)	1.30 ± 0.62	1.21 ± 0.45	*t* = 0.462	0.647
LDL (mmol/L)	2.61 ± 0.46	2.62 ± 0.49	*t* = −0.110	0.913
HDL (mmol/L)	1.38 ± 0.27	1.34 ± 0.24	*t* = 0.456	0.651
HbA1c (%)	7.87 ± 2.12	5.55 ± 0.32	*t* = 4.830	<0.001
(mmol/mol)	(62.50 ± 23.07)	(37.10 ± 3.60)		
FBG (mmol/L)	6.96 ± 1.72	4.84 ± 0.51	*t* = 5.158	<0.001
MMSE	29.05 ± 0.89	29.00 ± 0.94	*t* = 0.171	0.865
SAS	31.65 ± 5.91	31.42 ± 5.80	*t* = 0.122	0.904
SDS	33.85 ± 6.81	33.21 ± 7.18	*t* = 0.285	0.777

Values are mean ± standard deviation or Median (minimum, maximum) or number of subjects.

DBP = diastolic blood pressure; FBG = fasting blood glucose; HDL = high density lipoprotein; IES = inverse efficiency score; LDL = low density lipoprotein; MMSE = mini-mental state examination; SAS = self-rating anxiety scale; SBP = systolic blood pressure; SDS = self-rating depression scale; TC = total cholesterol; TG = Triglyceride.

**Table 2:** Brain regions showing significantly decreased intra-network functional connectivity in T2DM patients.

Region	IC	MNI coordinates			*T* *	Cluster size
		x	y	z		
Right lingual gyrus	10	24	−69	−15	−3.535	35
Left lingual gyrus	10	−12	−78	−9	−4.893	42
Left ventral lateral prefrontal cortex	19	−36	30	−3	−3.238	17
Left inferior parietal lobule	21	−54	−60	39	−3.753	20

^*^T value of two sample t-test (*P* < 0.05, Alphasim corrected). IC, independent component; MNI, Montreal Neurological Institute coordinate.
