# Supplementary material for: Altered brain activation and functional connectivity in working memory related networks in patients with type 2 diabetes: An ICA-based analysis
Source: Sci Rep. 2016 Mar 29;6:23767. doi: 10.1038/srep23767 (PMC4810460; doi:10.1038/srep23767)
Supplement: Supplementary Information [file srep23767-s1.doc]

**Altered brain activation and functional connectivity in working memory related networks in type 2 diabetes: an ICA based analysis**

Yang Zhang,^1#^ Shan Lu,^2#^ Chunlei Liu,^1^ Huimei Zhang,^1^ Xuanhe Zhou,^1^ Changlin Ni,^3^ Wen Qin,^1*^ Quan Zhang^1*^

^1^ Department of Radiology and Tianjin Key Laboratory of Functional Imaging, Tianjin Medical University General Hospital, Tianjin 300052, China.

^2^ Department of Radiology, Tianjin Medical University Metabolic Diseases Hospital, Tianjin, 300060, China.

^3^ Department of Cardiology, Tianjin Medical University Metabolic Diseases Hospital, Tianjin, 300060, China

**^*^ Corresponding authors:** Quan Zhang, Department of Radiology, Tianjin Medical University General Hospital, No. 154, Anshan Road, Heping District, Tianjin 300052, China. Phone: +862260363760; Fax: +862260362990; E-mail: zhangquan0912@163.com. Wen Qin, Department of Radiology, Tianjin Medical University General Hospital, No. 154, Anshan Road, Heping District, Tianjin 300052, China. Phone: +862260362026; Fax: +86-22-60362990; E-mail: wayne.wenqin@gmail.com

^#^ These authors contributed equally to this work.

***
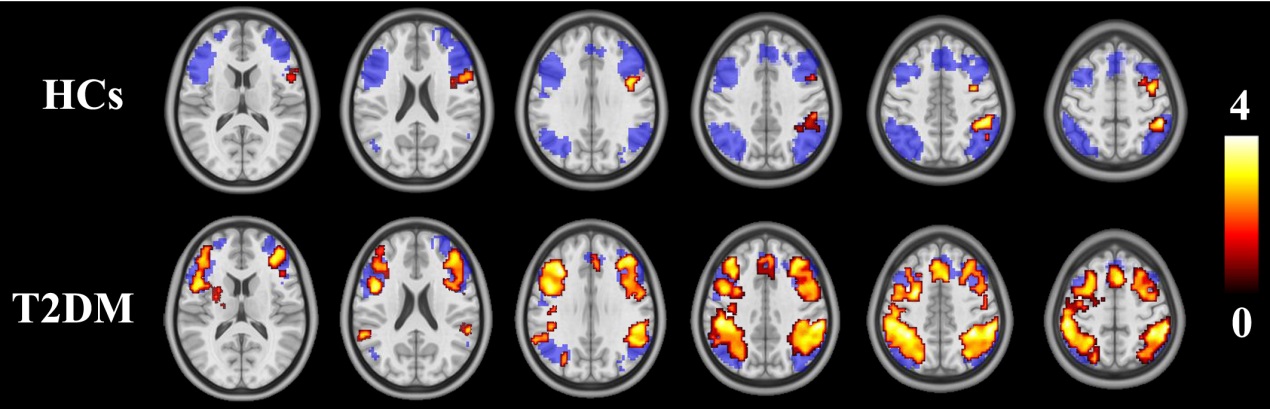
***

**Figure S1** Activated regions in T2DM patients and the HCs (One-sample *t*-test, *P* < 0.05, Alphasim corrected). The blue regions represent for a combined mask of IC19, IC21 and IC22.

***
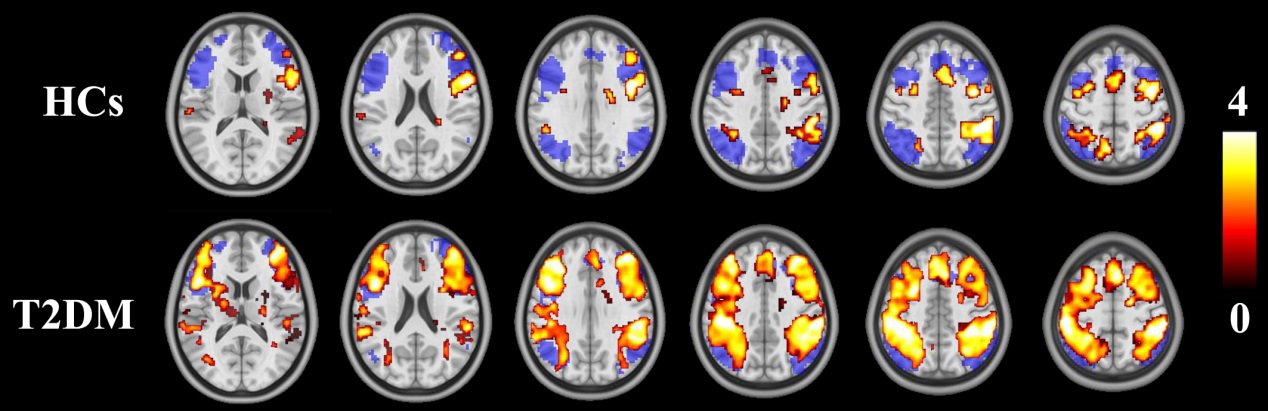
***

**Figure S2** Activated regions in T2DM patients and the HCs (One-sample *t*-test, *P* < 0.05, uncorrected). The blue regions represent for a combined mask of IC19, IC21 and IC22.


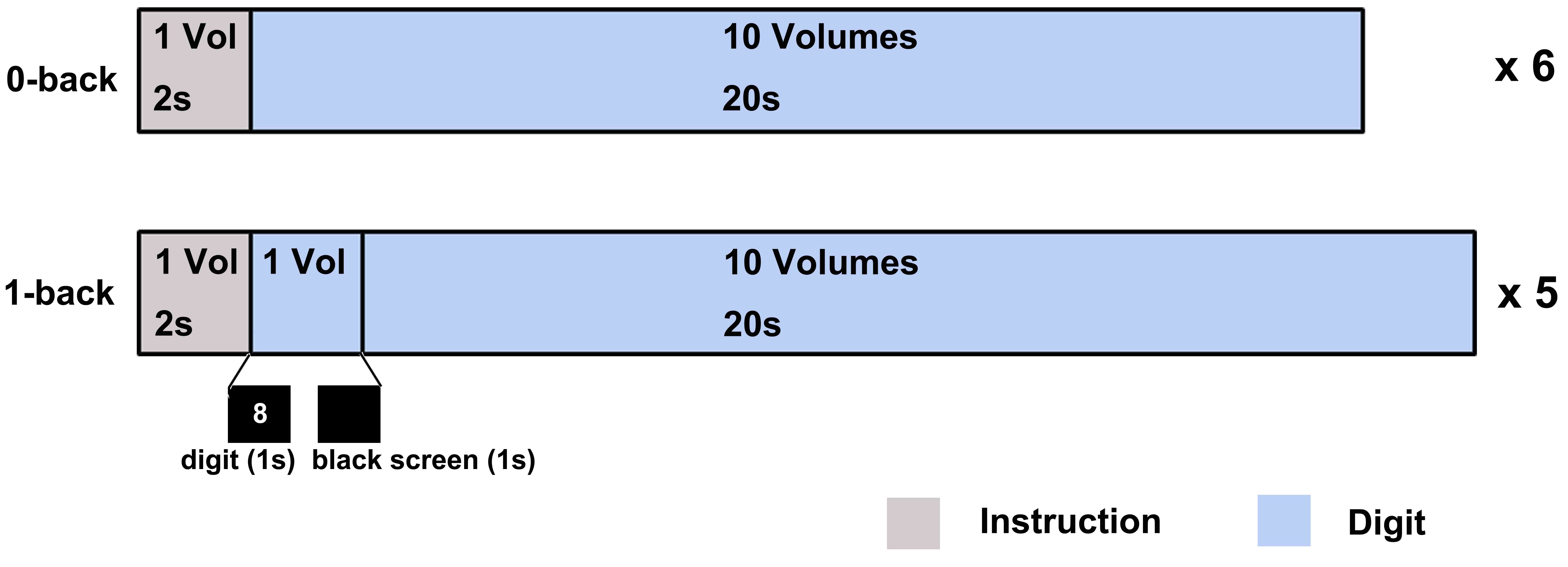


**Figure S3** The Timing diagrams of the n-back working memory task.

| **Table S1 2-back task performance of eight T2DM patients** | | | | | | | |
| --- | --- | --- | --- | --- | --- | --- | --- |
|  | Best performance in training | | | | Performance during MR scan | | |
|  | Accuracy rate (%) | | Response time (ms) | | Accuracy rate (%) | | Response time (ms) |
| Patient 1 | 46 | 955.87 | |  | |  | |
| Patient 2 | 20 | 874.30 | |  | |  | |
| Patient 3 | 60 | 942.76 | | 66 | | 1510.61 | |
| Patient 4 | 61.67 | 1245.45 | | 52 | | 1325.43 | |
| Patient 5 | unfinished |  | |  | |  | |
| Patient 6 | 42 | 766.86 | |  | |  | |
| Patient 7 | unfinished |  | |  | |  | |
| Patient 8 | 35 | 1021.11 | |  | |  | |
